# An exosome-related long non-coding RNAs risk model could predict survival outcomes in patients with breast cancer

**DOI:** 10.1038/s41598-022-26894-5

**Published:** 2022-12-24

**Authors:** Pengjun Qiu, Qiaonan Guo, Jianqing Lin, Kelun Pan, Jianpeng Chen, Mingji Ding

**Affiliations:** Department of Breast and Thyroid Surgery, The Second Affiliated Hospital of Fujian Medical University, No.950 Donghai Street, Quanzhou, China

**Keywords:** Breast cancer, Prognostic markers, Genetic markers, Tumour immunology

## Abstract

Breast cancer (BC) is one of the most frequent malignancies among women worldwide. Accumulating evidence indicates that long non-coding RNA (lncRNA) may affect BC progression. Exosomes, a class of small membrane vesicles, have been reported to promote tumor progression through transporting proteins, mRNAs, lncRNAs and some other small molecules. However, the interaction between exosome-related lncRNAs and the microenvironment of malignancies is unclear. Hence, we proceeded to investigate the relationship between exosome-related lncRNAs and BC microenvironment. 121 exosome-associated genes were extracted from ExoBCD database. Then, the Pearson analysis was used to screened out the exosome-related lncRNAs. After that, 15 exosome-related differentially expressed lncRNAs were identified by the correlation with BC prognosis. According to the sum of the expression of these 15 lncRNAs, extracted from The Cancer Genome Atlas, and the regression coefficients, an exosome-related lncRNAs signature was developed by using Cox regression analysis. With the median risk score of the training set, the patients in training and validation sets were separated to low-risk group and high-risk group. Subsequently, the lncRNA–mRNA co-expression network was constructed. The distinct enrichment pathways were compared among the different risk groups by using the R package clusterProfiler. The ESTIMATE method and ssGESA database were adopted to study the ESTIMATE Score and immune cell infiltration. Eventually, the expression of immune checkpoint associated genes, microsatellite instable and the immunophenoscore were further analyzed between different risk groups. Different risk groups exhibited different prognosis, with lower survival rate in the high-risk group. The differentially expressed genes between the different risk groups were enriched in biological processes pathways as well as immune responses. BC patients in high-risk group were identified with lower scores of ESTIMATE scores. Subsequently, we noticed that the infiltrating levels of aDCs, B cells, CD8+ T cells, iDCs, DCs, Neutrophils, macrophages, NK cells, pDCs, Tfh, T helper cells, TIL and Tregs were obvious elevated with the decreased risk score in training and validation cohorts. And some immune signatures were significantly activated with the decreased risk score in both cohorts. Eventually, the exosome-associated lncRNAs risk model was demonstrated to accurately predict immunotherapy response in patients with BC. The results of our study suggest that exosome-related lncRNAs risk model has close relationship with prognosis and immune cells infiltration in BC patients. These findings could make a great contribution to improving BC immunotherapy.

## Introduction

As the most diagnosed malignancy, BC was considered as the primary cause of female death around word. According to 2011–2017 data from the SEER database (https://seer.cancer.gov/), the 5-years relative survival of patients with BC up to 90.3%. BC accounted for about 14.8% of all new malignancy cases, and the mortality rate was close to 7.2% in United States in 2021. Thanks to the improvement of diagnosis and treatment therapy, the mortality rate of BC has been decreased year by year. However, BC is a highly heterogeneous cancer, and the clinical outcomes of patients with BC is mostly correlated with immunity^1^. Developing tumor metastasis and invasion are main causes of death in BC patients. Hence, it is urgent to screen reliable prognostic indicators and therapeutic targets for BC to guide correct individual treatment strategies.

Varieties of factors are involved in the development and metastasis of BC, including long noncoding RNAs (lncRNAs)^2^. lncRNAs are a class of transcript RNAs that are longer than 200 nucleotides^3^. As vital immunity related regulators, lncRNAs have critical functions in distinct stage of cancer immunity, including antigen presentation, immune activation, and immune cell infiltration^4–6^. Moreover, lncRNAs are suggested highly potential to be promising diagnostic^7^, therapeutic agents^8^, and prognostic biomarkers in BC^9^. However, the functions of lncRNAs and their mechanisms in BC progression is still unclear. Exosomes are a type of homogenous membrane vesicles that carry a variety of small molecules including proteins, miRNAs and lncRNAs, can be collected in body fluids with an average size of 40-150nm^10–12^. Accumulating evidence showed that exosomes play a crucial role in metastasis and recurrence of tumor^13,14^. In addition, some studies indicated that exosomal lncRNAs are abundant and more stable in the body fluids, and they play an important role in promoting tumor proliferation, migration, invasion, and drug resistance^9,14–19^. Hence, we hypothesized that lncRNAs affect BC progression through exosomes related immunity response.

In current study, a dataset of lncRNA expression in BC from The Cancer Genome Atlas (TCGA) were analyzed and the prognostic lncRNAs associated with exosomes were screened out. After that, a 15 exosome-related lncRNA signature was identified with the highly potential ability to predict the survival outcomes of BC patients.

## Methods

### Workflow

**Figure 1:**
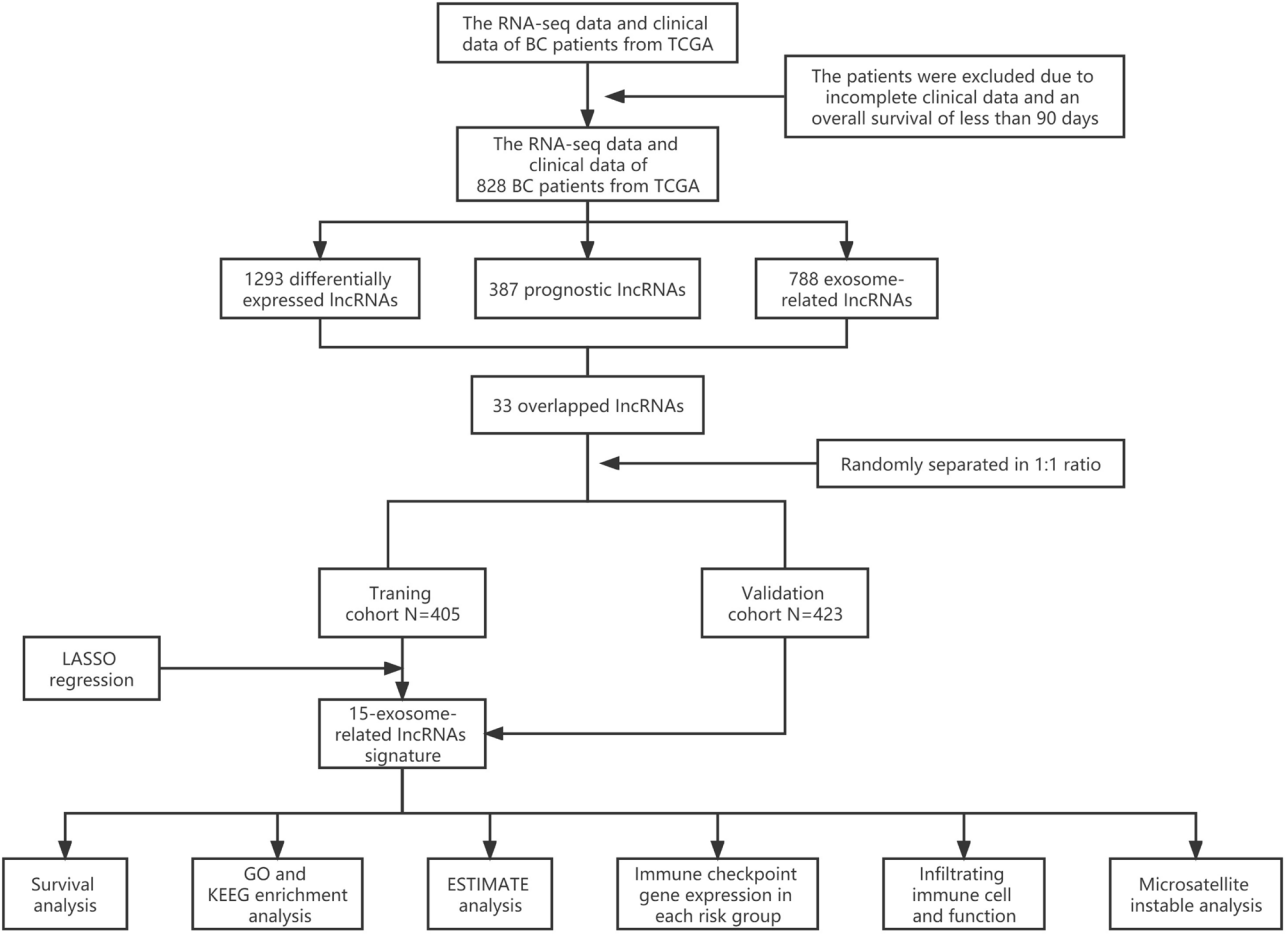
Analysis flow chart.

A combination of several methods was employed to establish a 15-lncRNA risk model and investigated the potential mechanisms of these lncRNAs affect the prognostic outcomes of BC (Fig. 1).

### Data acquisition

RNA-sequencing expression date and clinical information of BC patients were downloaded from TCGA (http://cancergenome.nih.gov/). The conditions of eligible samples were as follows: (1) samples have both completed clinical prognostic information as well as transcriptome expression data; (2) samples excised from primary tumors. The exclusion criteria were as follows: (1) sample with an overall survival (OS) of less than 90 days; (2) samples with incomplete clinical information. Consequently, after batch normalization, 828 BC patients from the TCGA with complete clinical data were enrolled for subsequent analysis. 121 exosome-associated genes were collected from ExoBCD database (https://exobcd.liumwei.org/) and presented in Supplemental Table 1.

### Identification of exosome-related lncRNAs

A total of 3158 lncRNAs were involved in current study, with the one whose average expression value less than 1 were excluded. Subsequently, the Pearson correlation coefficients were calculated to identify the relevance of exosome-associated gene expression and correspond lncRNA. After that, the exosome-associated lncRNAs were selected based on the criteria that *p* < 0.01 and |R|> 0.4.

### Establishment of the prognostic exosome-related lncRNAs signature

The differentially expressed lncRNAs between normal breast samples and BC samples were identified by the “edge R” package. The lncRNAs that met cutoff criteria of |log2fold change (FC)|> 1 and *p* value < 0.05 were regarded as differentially expressed lncRNAs, visualized by volcano plot. Subsequently, univariate Cox regression analysis of OS was conducted to screen out lncRNAs associated with prognosis. After that, the overlapping lncRNAs of exosome-related lncRNAs, prognostic lncRNAs and differentially expressed lncRNAs were selected as the candidate lncRNAs to establish the prognostic exosome-associated lncRNAs risk model. A total of 828 patients with BC were randomized in a 1:1 ratio to either a training set or a validation set to establish and validate the signature of lncRNAs associated with exosomes. To reduce redundant lncRNAs and avoid model over-fitting, the least absolute shrinkage and selection operator (LASSO) Cox regression model was established to determine all independent prognostic lncRNAs. Consequently, 15 optimal exosome-related lncRNAs were selected for the construction of prognostic risk model. The risk score of each patient on the basis of this risk model was calculated through the normalized exosomal lncRNAs expression levels and correspond coefficients. The calculation formula was as follows: Risk score = ${\sum }_{i=1}^{n}(Ex{p}_{i}*Co{e}_{i})$. (N = 15, $Ex{p}_{i}$ indicated the expression level for each exosome-associated lncRNA, and $Co{e}_{i}$ indicated the correspond Cox regression coefficient.) As a result, patients in training set were separated into high-risk and low-risk groups according to the median risk score of training set. Survival analysis was conducted between the different risk groups by the “survminer” R package and time-dependent ROC curve analysis was further conducted to assess the forecast accuracy of the risk model. To further validate this prognostic signature, the risk score of each BC patient was calculated in validation set according to the same formula, and the patients were separated to low-risk and high-risk groups according to the same cut-off value of training set. Finally, the survival analysis and the time-dependent ROC curve analysis were conducted in validation cohort. As consequence, underwent univariate and multivariate COX regression analysis, the risk score was testified as the independent prognostic element for BC patients.

### Construction of the lncRNA-mRNA co-expression network

The Cytoscape software was employed to construct the mRNA-lncRNA co-expression network to further identify the relevance of the selected exosome-related lncRNAs and their corresponding mRNAs. Besides, the degree of correlation between them was visualized by the Sankey diagram.

### Functional enrichment analysis

Gene Ontology (GO) enrichment analysis and Kyoto Encyclopedia of Genes and Genomes (KEGG) pathway analysis were conducted in the differentially expressed genes (DEGs) between low-risk and high-risk groups with the R clusterProfiler package to elucidate the potential signaling pathway. Biological process (BP), cellular component (CC), and molecular function (MF) are contained in GO terms. *p* value < 0.05 was considered statistically significant in functional enrichment analysis.

### Correlation assessment of risk score and tumor immune environment characterization

Several analyses were applied to distinguish the difference of tumor immunity microenvironment (TIME) between different risk groups. Estimation of Stromal and Immune cells in Malignant Tumor tissues using expression (ESTIMATE) algorithm^20^ was employed to calculate the rate of the immune-stromal component in TIME via package “estimate” in R, including Stromal Score, Immune Score, and ESTIMATE Score. The respective scores implied the proportion of the corresponding components in the TIME. Subsequently, the single-sample gene set enrichment analysis was performed by “GSEAbase” package of R to identify the enrichment of immune function associated gene sets.

### Correlation between immune checkpoint blockade treatment and exosome-related lncRNAs risk signature

According to the reported research, the expression levels of immune checkpoint genes could be related to clinical outcomes of immune checkpoint inhibitors. Hence, 5 key genes of immune checkpoint blockade treatment associated genes were selected in current study: *PD-1*, *PD-L1*, *LAG3*, *CTLA-4* and *TIM3*. The expression levels of the 5 genes were calculated respectively in high and low risk groups and the results were plot by the “GGPUBR”, “ggplot2”, and “ggExtra” R packages.

### Prediction of immunotherapy response by exosome-related lncRNAs risk signature in BC patients

As for the prediction of immunotherapy response, the transcriptional expression of significant mismatch repair genes was calculated and compared in tumor samples, including *MSH2*, *MSH6*, *MLH1*, and *PMS2*. Moreover, The Cancer Immunome Atlas (TCIA) database (https://tcia.at/home) was employed to calculate the immunophenoscore (IPS) in each sample, which served as a favorable predictor of response to *CTLA-4* and *PD-1*. The IPS of BC patients was extracted from TCIA and obtained without bias by considering 4 categories of immunogenicity- determining genes: effector cells, immunosuppressor cells, MHC molecules, and immune modulators. This procedure was conducted by assessing gene expression in four cell types and the IPS was calculated with a range of 0–10 based on the z-score for gene expression of representative cell types^21^. For each category, a sample-wise z-score was calculated. After that, the weighted average z-score was computed by averaging the z-scores in the respective categories to obtain 4 values, with the sum of the weighted average z-scores of the 4 categories being identified as the IPS^22^. Finally, the IPS in high and low risk groups were analyzed to identify the correlation among IPS and risk scores. *p* < 0.05 was considered statistically significant.

### Statistical analysis

Statistical analyses were conducted through R software (Version 4.0.5) (https://www.r-project.org/). Chi-square tests were performed on the correlation of clinicopathological variables in the high-risk and low-risk groups of BC patients. Pearson correlation analysis was adopted to identify the relevance of the exosome-related gene expression and correspond lncRNA. The Wilcoxon test was used to check the differences between the two sets of variables. Kaplan–Meier curve was applied to analysis the survival data. The univariate and multivariate Cox regression analyses were employed to evaluate the independent prognostic factors. *p* < 0.05 was regarded statistically significant.

## Results

### Data source and processing

Initially, a total of 3158 lncRNAs were obtained by analyzing the RNA-seq data of 828 BC samples and 112 normal breast samples from TCGA. Besides, the correspond clinical information of 828 patients were extracted from TCGA. And the basic characteristics of BC patients in the training and validation sets were presented in Table 1. Besides, the exosome-related gene set was downloaded from ExoBCD database, containing 121 genes participate in exosome-related regulation, among which, 117 genes were ascertained in TCGA database. After that, the expression levels of 788 lncRNAs were shown correlated (|R|> 0.4 and *p* < 0.01) with exosome-related genes (Supplemental Table 2).

**Table 1:** Clinicopathological characteristics of BC patients in this study.

Variables	Training cohort (n = 405)		Validation cohort (n = 423)		*p* value
	No	%	No	%	
**Age**					
< 60	219	54.1	239	56.5	0.527
≥ 60	186	45.9	184	43.5	
**T**					
T1	114	28.1	120	28.4	0.554
T2	226	55.8	229	54.2	
T3	46	11.4	61	14.4	
T4	17	4.2	12	2.8	
Tx	2	0.5	1	0.2	
**N**					
N0	189	46.7	187	44.2	0.397
N1	143	35.3	145	34.3	
N2	35	8.6	53	12.5	
N3	29	7.2	32	7.6	
Nx	9	2.2	6	1.4	
**M**					
M0	333	82.2	360	85.1	0.384
M1	9	2.2	11	2.6	
Mx	63	15.6	52	12.3	
**Stage**					
Stage I	81	20.0	73	17.3	0.356
Stage II	226	55.8	225	53.2	
Stage III	79	19.5	106	25.0	
Stage IV	8	2.0	10	2.4	
Stage X	11	2.7	9	2.1	

### Identification of prognostic differentially expressed exosome-associated lncRNAs

**Figure 2:**
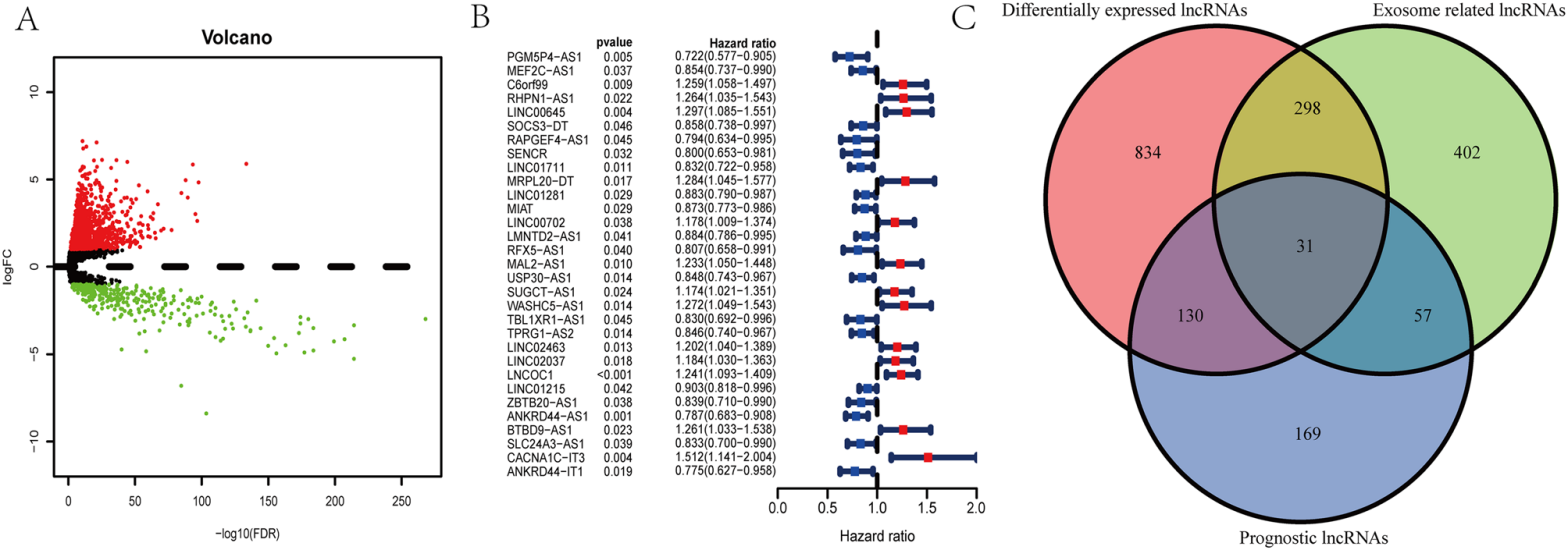
Identification of differentially expressed exosome-associated prognostic lncRNAs in BC patients. (**A**) Volcano plot of differentially expressed lncRNAs. Each green dot represents a downregulated gene and each red dot represents an upregulated gene. (**B**) The forest plot presented the HR (95% CI) and *p* value of selected prognostic lncRNAs through univariate Cox regression analysis. (**C**) Venn diagram to distinguish the overlapped lncRNAs of exosome-related lncRNAs differentially expressed lncRNAs, and prognostic lncRNAs.

A total of 1293 differentially expressed lncRNAs were identified by “edge” R package in BC patients, among which, 322 were down-regulated and 971 were up-regulated (Fig. 2A). Follow by univariate Cox regression analysis, 387 prognostic lncRNAs were confirmed related to patients’ OS (*p* < 0.05), provided in Supplemental Table 3. Consequently, 31 overlapping lncRNAs (Fig. 2B) were selected as candidate lncRNAs from differentially expressed lncRNAs, exosome-associated lncRNAs and prognostic lncRNAs via Venn diagrams (Fig. 2C).

### Construction and validation of an exosome-related prognostic model

**Figure 3:**
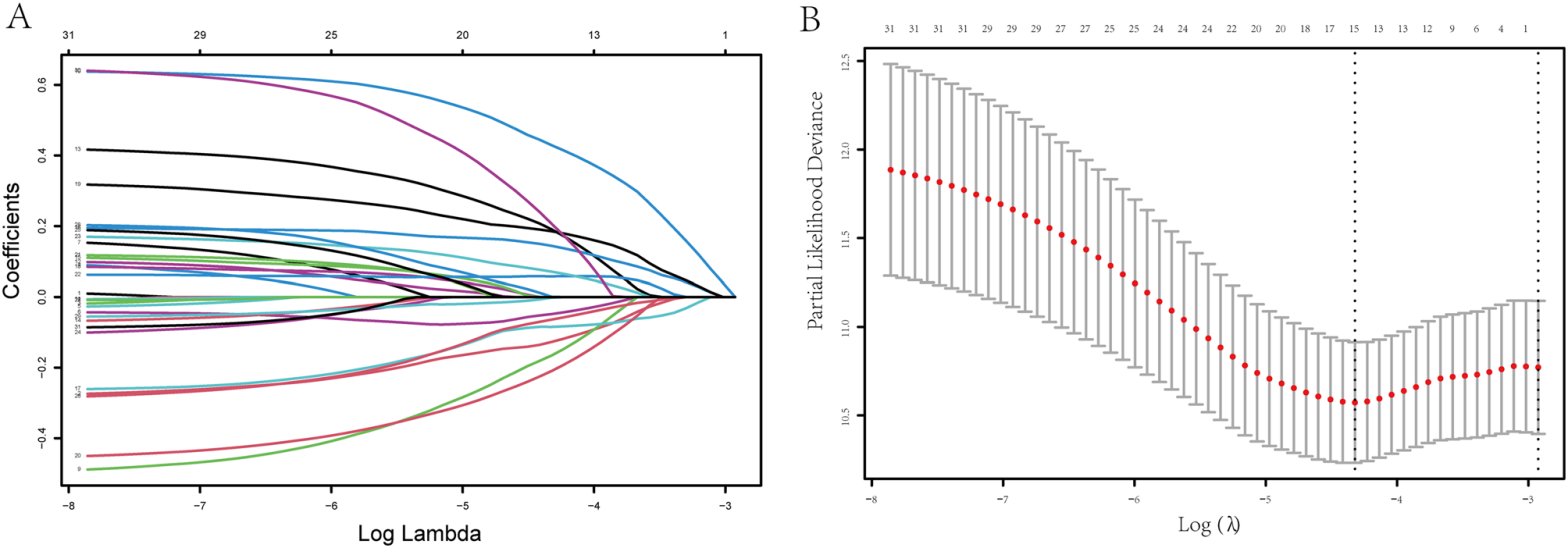
Screening for exosome-related lncRNAs and their relationship with patient prognosis by LASSO Cox regression analysis. (**A**) LASSO coefficient profiles of 31 lncRNAs with *p* < 0.01. (**B**) fivefold cross-validations result which determined best values of the penalty parameter λ.

Thirty-one lncRNAs mentioned were subjected to LASSO regression analysis to avoid overfitting of the predictive signal. Moreover, the LASSO coefficient profiles of the 31 lncRNAs were provided (Fig. 3A) and fivefold cross-validation results were generated to determine best values of the penalty parameter λ (λ = 0.01328404) (Fig. 3B). Finally, a total of 15 exosome-related lncRNAs (*MEF2C-AS1*, *SOCS3-DT*, *LINC01711*, *MRPL20-DT*, *LINC00702*, *MAL2-AS1*, *USP30-AS1*, *WASHC5-AS1*, *TBL1XR1-AS1*, *LINC02463*, *LINC02037*, *ZBTB20-AS1*, *BTBD9-AS1*, *SLC24A3-AS1*, *CACNA1C-IT3*) were obtained for follow study. Accordingly, 15-lncRNA prognostic signature to assess the OS of BC patients was established based on the expression of 15 vital lncRNAs and their regression coefficients as follows: Risk score = (− 0.127 × expression level of *MEF2C-AS1*) + (− 0.048 × expression level of *SOCS3-DT*) + (− 0.165 × expression level of *LINC01711*) + (0.429 × expression level of *MRPL20-DT*) + (0.190 × expression level of *LINC00702*) + (0.144 × expression level of *MAL2-AS1*) + (− 0.084 × expression level of *USP30-AS1*) + (0.185 × expression level of *WASHC5-AS1*) + (− 0.209 × expression level of *TBL1XR1-AS1*) + (0.058 × expression level of *LINC02463*) + (0.071 × expression level of *LINC02037*) + (− 0.064 × expression level of *ZBTB20-AS1*) + (0.0005 × expression level of *BTBD9-AS1*) + (− 0.002 × expression level of *SLC24A3-AS1*) + (0.206 × expression level of *CACNA1C-IT3*). As a result, the patients were separated to high-risk and low-risk cohorts on the basis of the median risk score.

**Figure 4:**
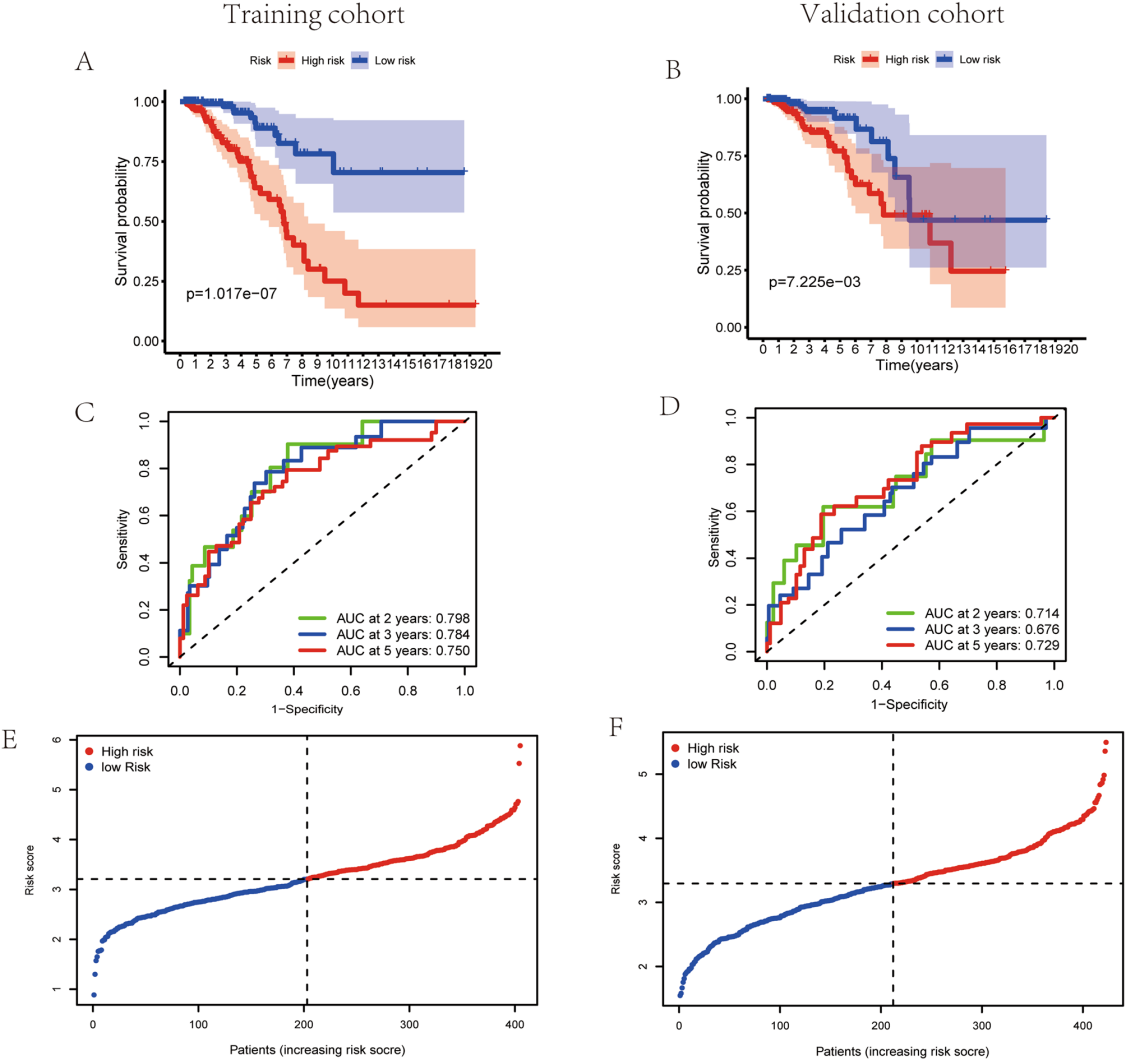
Evaluation of exosome-associated lncRNA risk model in training set and validation set. (**A**,**B**) Kaplan–Meier survival curves for BC patients in training set (**A**) and validation set (**B**), shown that the OS of the low-risk groups were significantly higher than the OS of the high-risk groups, respectively (*p* = 1.017E−07 and *p* = 4.375E−03, respectively). (**C**,**D**) ROC curve analysis of the accuracy of the model to predict patient prognosis at 2, 3 and 5 years in the training set (**C**) and the validation set (**D**). The distribution and median value of the risk score in training set (**E**) and the median risk score of training set was set as the cut-off value of high and low risk groups in validation set (**F**).

As indicated in Fig. 4A, patients with high-risk score are demonstrated worse survival rates in the training set by the Kaplan–Meier curves (*p* < 0.01). After that, a time-dependent ROC analysis was performed at 2, 3 and 5 years to assess the prognostic accuracy of the risk score. Consequently, the identified prognostic features were demonstrated promising efficient in predicting OS in BC patients via the area under the curve (AUC) (AUC = 0.798, 0.784 and 0.750; at 2, 3 and 5 years, respectively, Fig. 4C). Similarly, 423 patients of validation cohort were enrolled and the risk score of each patient was calculated on the basis of the mentioned 15-lncRNA signature. As suggested in Fig. 4B, patients with high-risk score are manifested worse survival rates in validation set via the Kaplan–Meier curves (*p* < 0.01). Remarkably, the risk score had been validated robust predictive value for BC survival, presented in the time-dependent ROC analysis (AUC = 0.714, 0.676 and 0.729; at 2, 3 and 5 years, respectively, Fig. 4D). Hence, a 15-lncRNA signature was successfully established to predict prognostic outcomes of BC patients. Based on the median risk score of training set (Fig. 4E), patients in validation set were separated to high-risk or low-risk groups (Fig. 4F).

**Figure 5:**
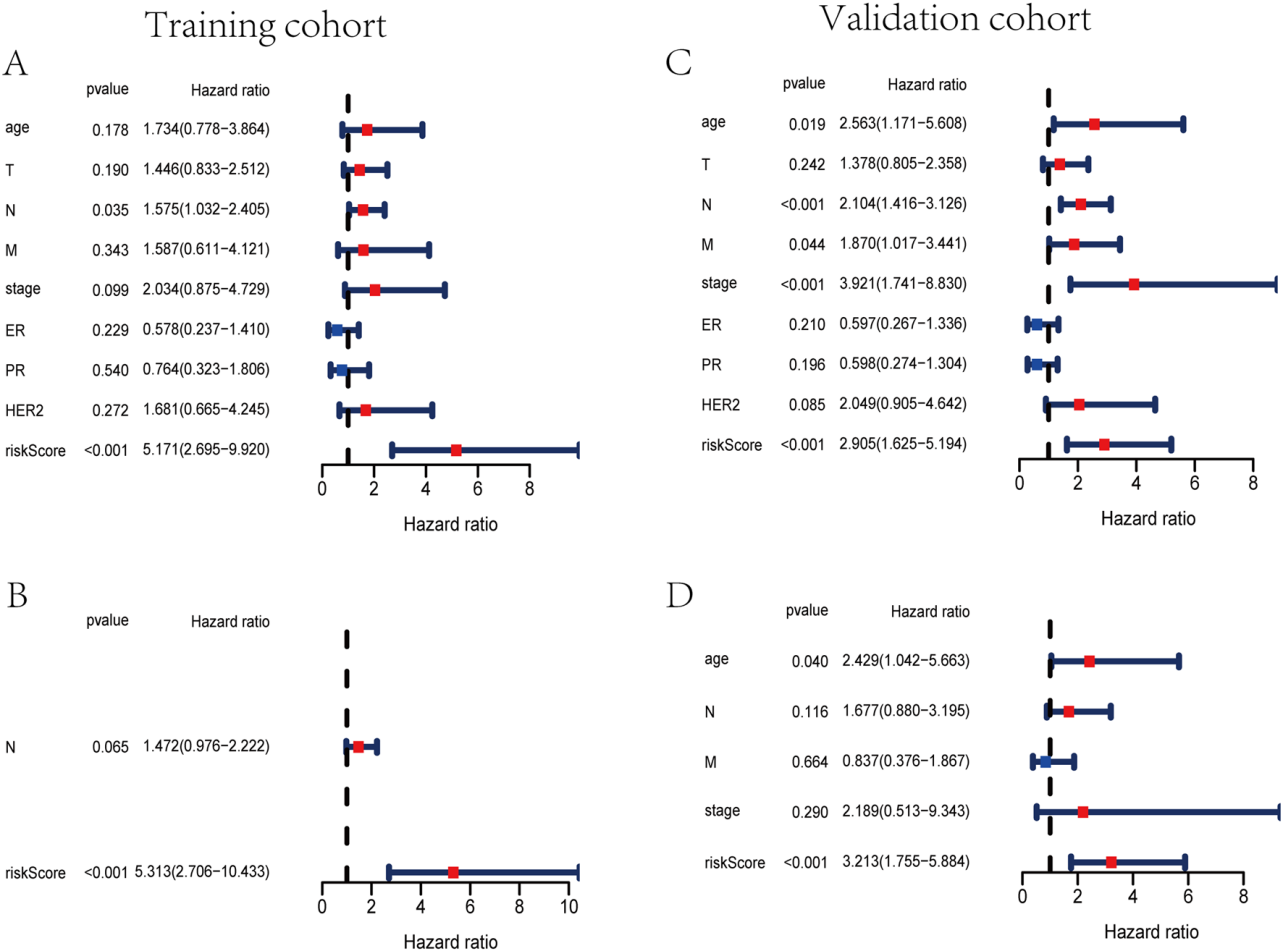
Assessment of exosome-associated lncRNAs risk score as the independent prognostic factor in BC. Univariate and multivariate Cox regression analysis of the risk model in the training cohort (**A**,**B**) and the validation cohort (**C**,**D**). (**A**) The forest plots for univariate Cox regression analysis indicated that risk score, and lymph node status were variables associated with prognostic risk in training set. (**B**) The forest plots for multivariate Cox regression analysis indicated that risk score was independent prognostic factors in training set. (**C**) The forest plots for univariate Cox regression analysis indicated that risk score, age, lymph node status, and AJCC stage were prognostic risk-related variables in validation set. (**D**) The forest plots for multivariate Cox regression analysis indicated that risk score and age were independent factors associated with prognosis in validation set.

To further evaluate the efficacy of the 15-lncRNA risk model as an independent prognostic factor for BC patients, univariate and multivariate Cox regression analysis were conducted between the risk score and clinical characteristics. The outcomes of univariate Cox regression analysis revealed that the risk score was an independent prognostic factor for BC patients in training and validation cohorts (Training set: HR 5.171, 95% CI 2.695–9.920, *p* < 0.001; Validation set: HR 2.905, 95% CI 1.625–5.194, *p* < 0.001, respectively) (Fig. 5A and Fig. 5C). After that, multivariate Cox regression analysis was performed to adjust for some confounding factors. The results reveled that the risk score remained an independent predictor of OS for BC patients (Training set: HR 5.313, 95% CI 2.706–10.433, *p* < 0.001; Validation set: HR 3.213, 95% CI 1.755–5.884, *p* < 0.001, respectively) (Fig. 5B and Fig. 5D). Finally, based on the 15-lncRNA signature, the difference of risk scores among subtypes of BC were analyzed, including Luminal A, Luminal B, basal and Her2-positive^23^. The Supplemental Fig. 1 indicated the risk score of Luminal A BC subtype was lower than those of other three subtypes (Luminal A vs basal, *p* < 0.05; Luminal A vs Her2+, *p* < 0.05; Luminal A vs Luminal B, *p* < 0.05).

### Establishment of the lncRNA–mRNA co-expression network

**Figure 6:**
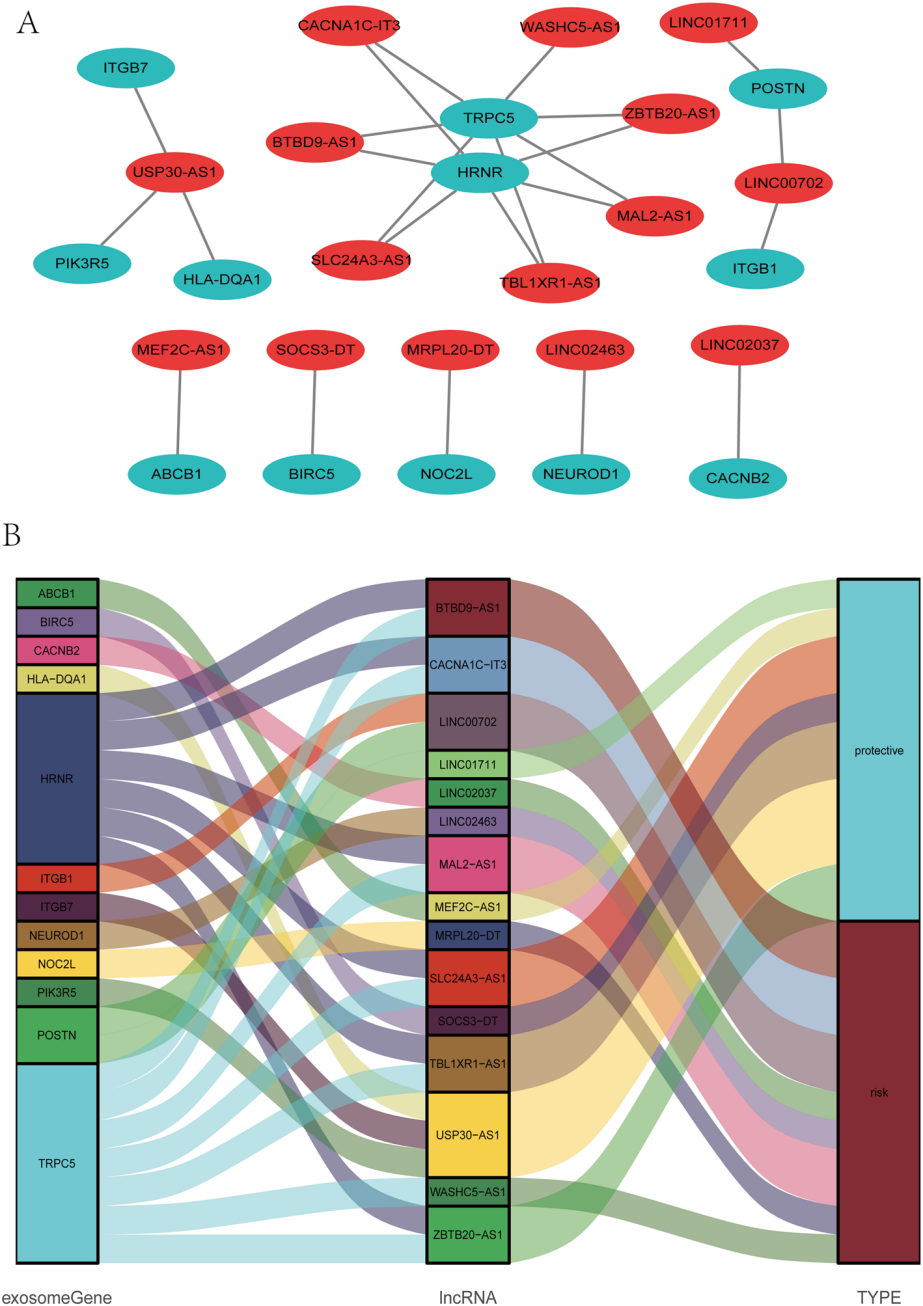
Construction of a LncRNA-mRNA co-expression network. (**A**) Diagrammatic plot showed the lncRNA-mRNA co-expression network included 24 lncRNA-mRNA pairs developed with 15 exosome-related risk lncRNAs and 12 mRNAs. The exosome-related mRNAs were represented as blue balloons and the exosome-related lncRNA were represented as red balloons. (**B**) Sankey diagram indicated the correlation among 15 exosome-related risk lncRNAs, 12 mRNAs, and risk types (risk or protective).

A lncRNA-mRNA co-expression network was established included 24 lncRNA-mRNA pairs to investigate the potential roles of the 15 exosome-related lncRNAs in BC (Fig. 6A). The Sankey diagram not only presented the association between 15 exosome-associated lncRNAs and targeted mRNAs, but also presented the correlation between exosome-associated lncRNAs and the risk types consisted of risk or protective factors (Fig. 6B).

### KEGG and GO functional enrichment analysis

**Figure 7:**
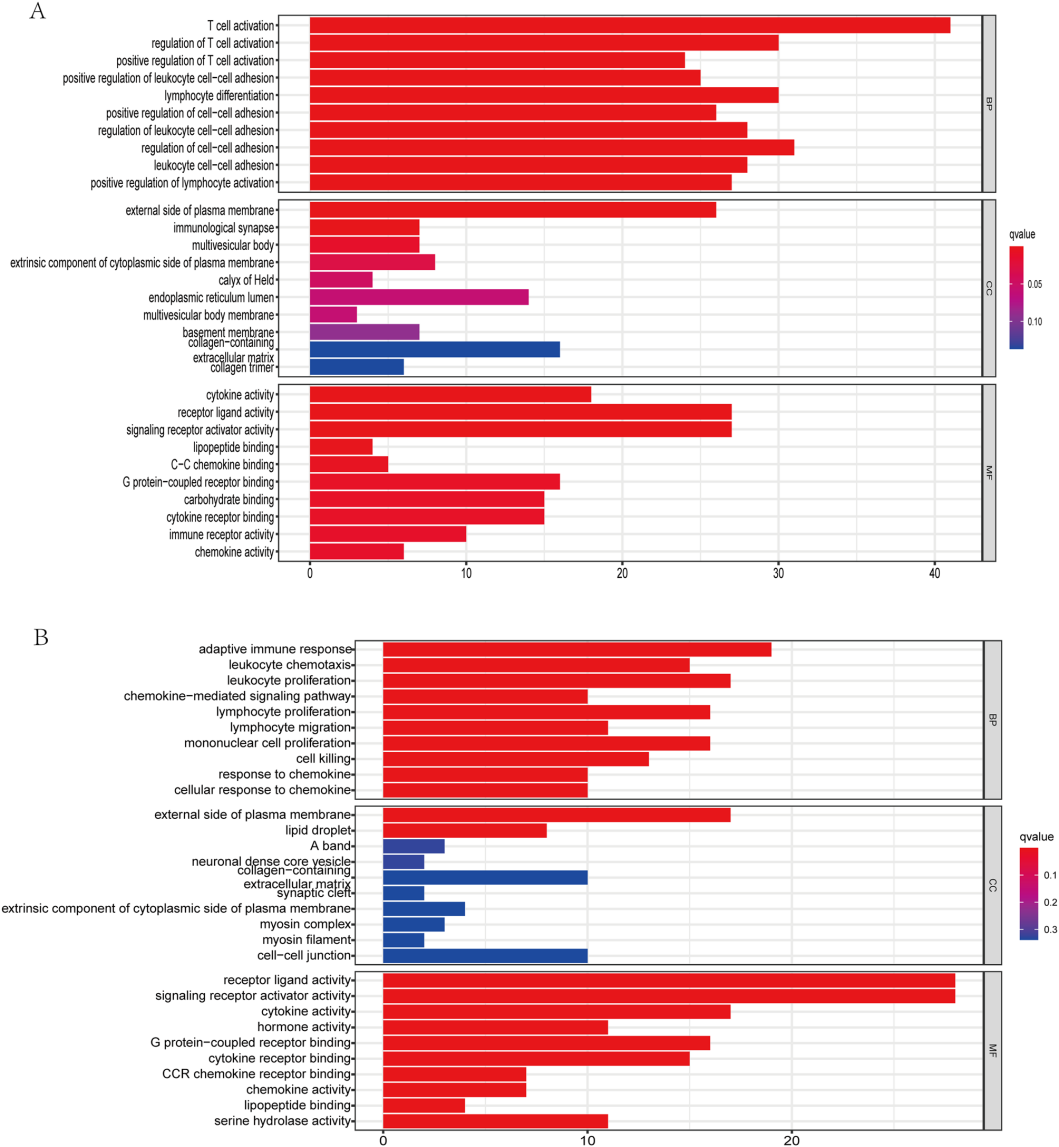
Representative results of GO enrichment analysis in training and validation cohorts. The outcomes of BP enrichment, CC enrichment, and MF enrichment of DEGs between high and low risk sets in training cohort (**A**) and validation cohort (**B**).

**Figure 8:**
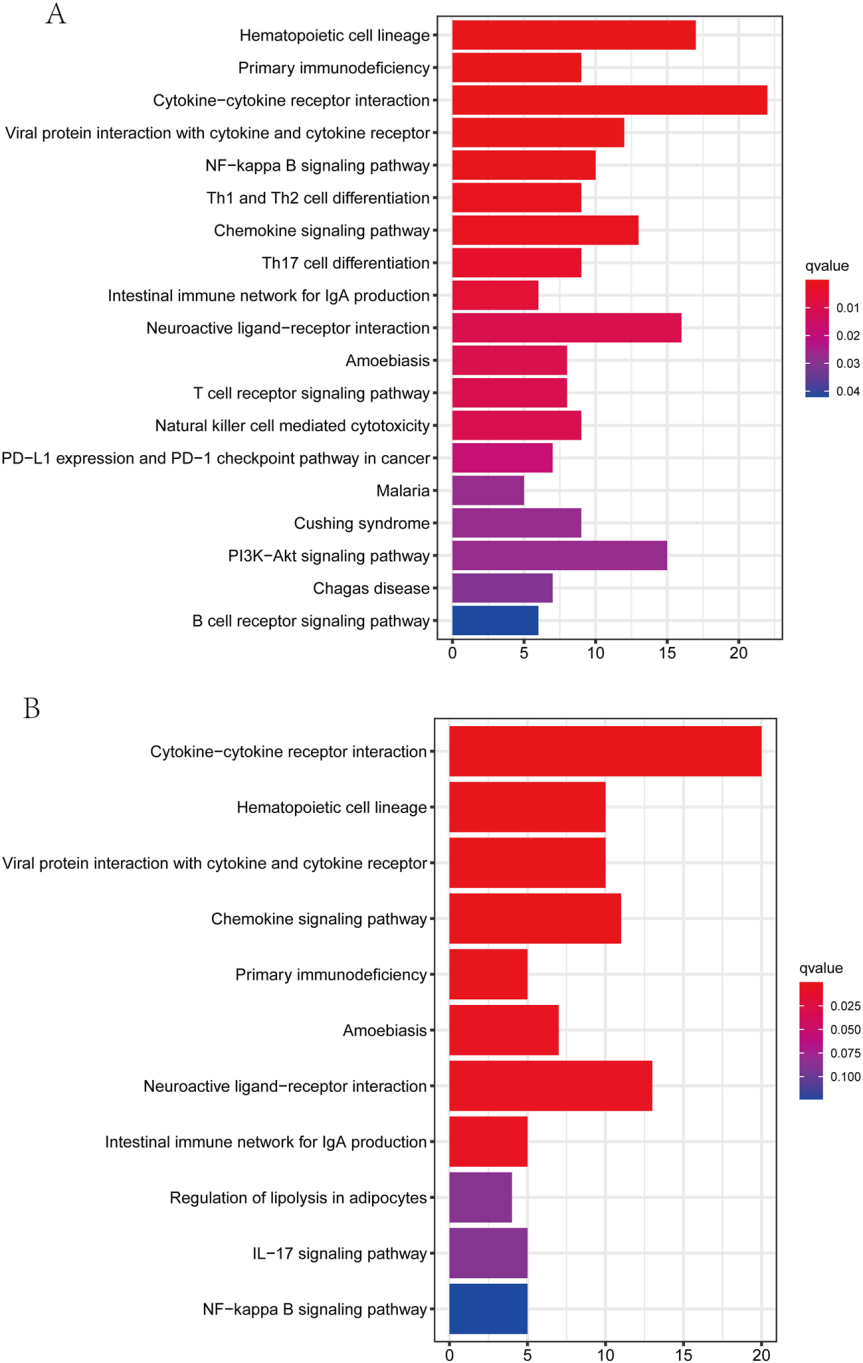
Representative results of KEGG enrichment analysis in training and validation cohorts. The outcomes of KEGG pathways analysis of DEGs between high and low risk sets in training cohort (**A**) and validation cohort (**B**).

GO and KEGG analysis were adopted to identify the biological functions and signaling pathways associated with the exosome-related risk score. The first 30 GO terms are presented in Fig. 7, included CC, BP, and MF. In addition, the 19 and 11 KEGG pathways in training and validation cohorts are displayed in Fig. 8, respectively. We found that majorities of GO terms and KEGG pathways were identified associated with immune activation and response.

### Correlation of ESTIMATE score and exosome-related risk model

**Figure 9:**
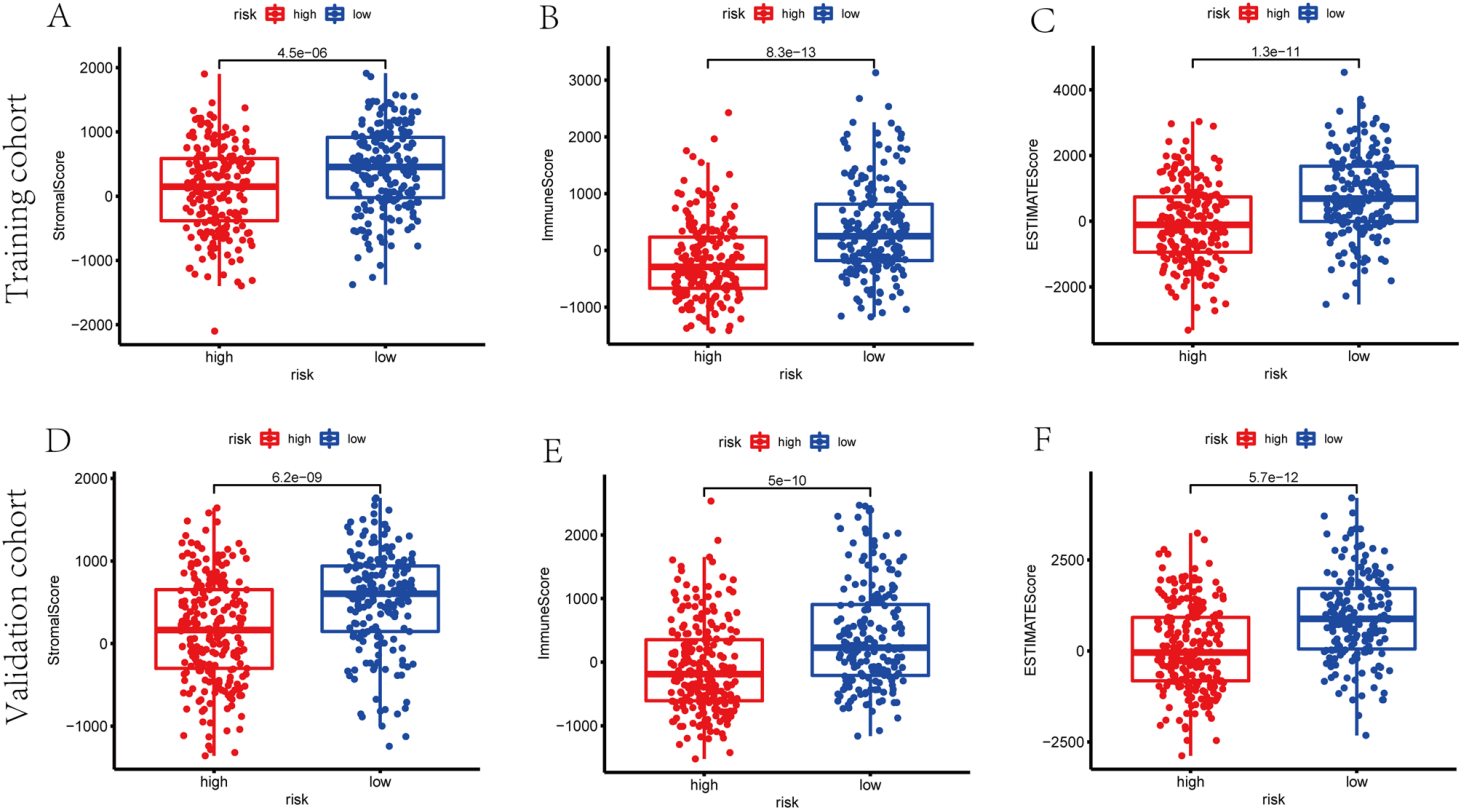
The scatter plot showed that the distributions of immune scores, stromal scores, and ESTIMATE scores were different between high- and low- risk groups in training set (**A**–**C**) and validation set (**D**–**F**).

To analyze the tumor microenvironment (TME) landscape and the overall degree of immune infiltration, we calculated the ESTIMATE score of each sample by ESTIMATE algorithm. As a result, patients in high-risk group were demonstrated with lower stromal scores, immune scores and ESTIMATE scores in training and validation cohorts (*p* < 0.01) (Fig. 9).

### Tumor immune environment characterization of BC

**Figure 10:**
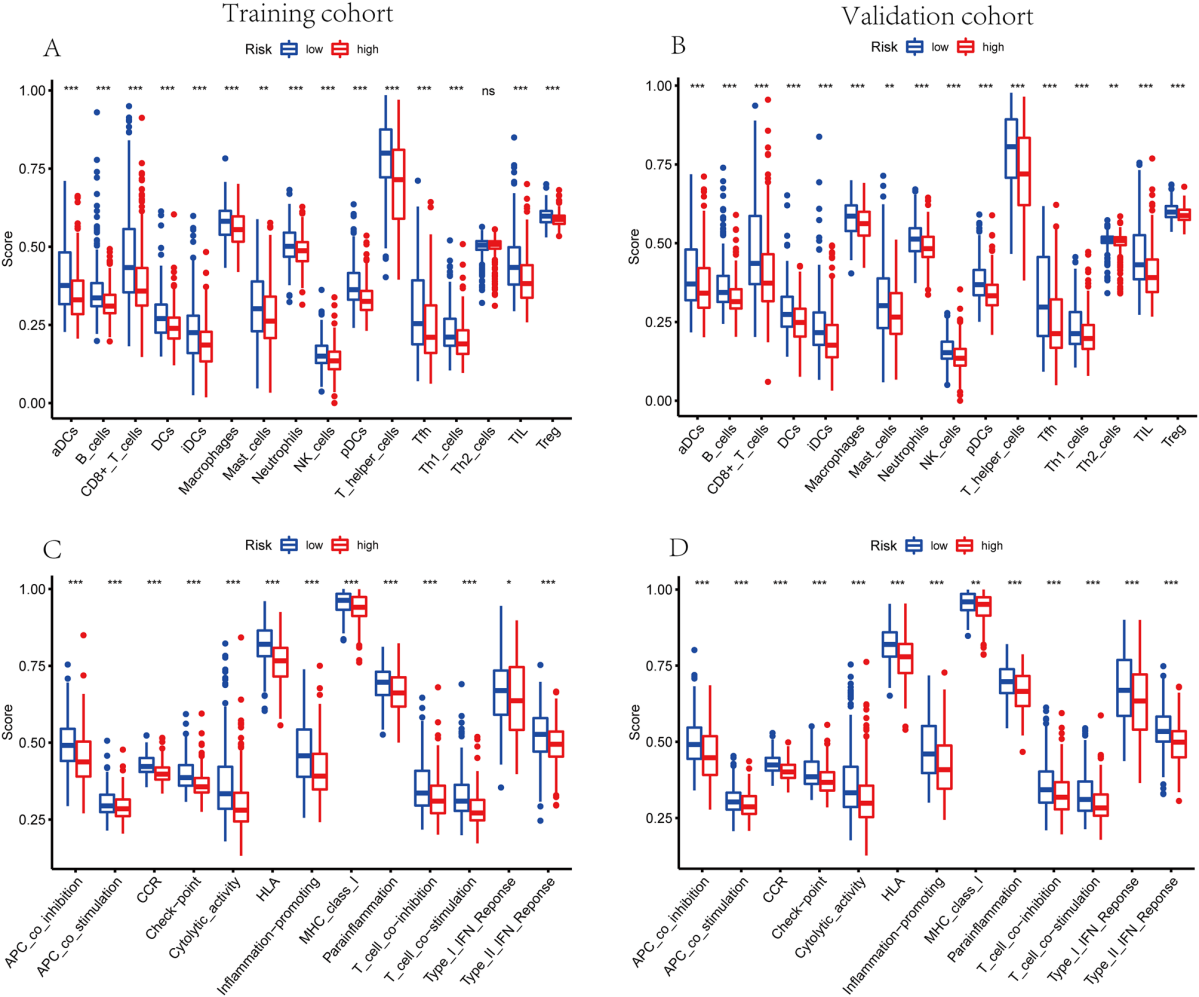
Correlation of exosome-related lncRNAs risk score with TIME characterization of BC. (**A**,**B**) Differences of infiltrating immune cell subsets and levels between low- and high- risk sets of training cohort (**A**) and validation cohort (**B**). (**C**,**D**) Distinguishing of enrichment of immune-associated signatures between high- and low- risk groups of training cohort (**C**) and validation cohort (**D**) (* indicated *p* < 0.05, ** indicated *p* < 0.01, *** indicated *p* < 0.001).

The GO and KEGG enrichment analysis indicated that the DEGs between the different risk groups were typically enriched in the pathways related to immunity. Therefore, we explored the distinction of immune signatures between high- and low- risk groups. As presented in ssGSEA results, the infiltrating levels of B cells, CD8+ T cells, aDCs, DCs, iDCs, pDCs, Neutrophils, macrophages, NK cells, T helper cells, Tfh, Th1 cells, TIL and Tregs were remarkably elevated with the decreased risk score in training and validation sets (Fig. 10A,B). Besides, some immune signatures were remarkably activated with the decreased risk score in both cohorts, for instance APC co-inhibition, APC co-stimulation, CCR, checkpoint, HLA, cytolytic activity, inflammation promoting, para-inflammation, T cell co-stimulation, T cell co-inhibition, and IFN response type II (Fig. 10C,D).

### Prediction of immunotherapy response

**Figure 11:**
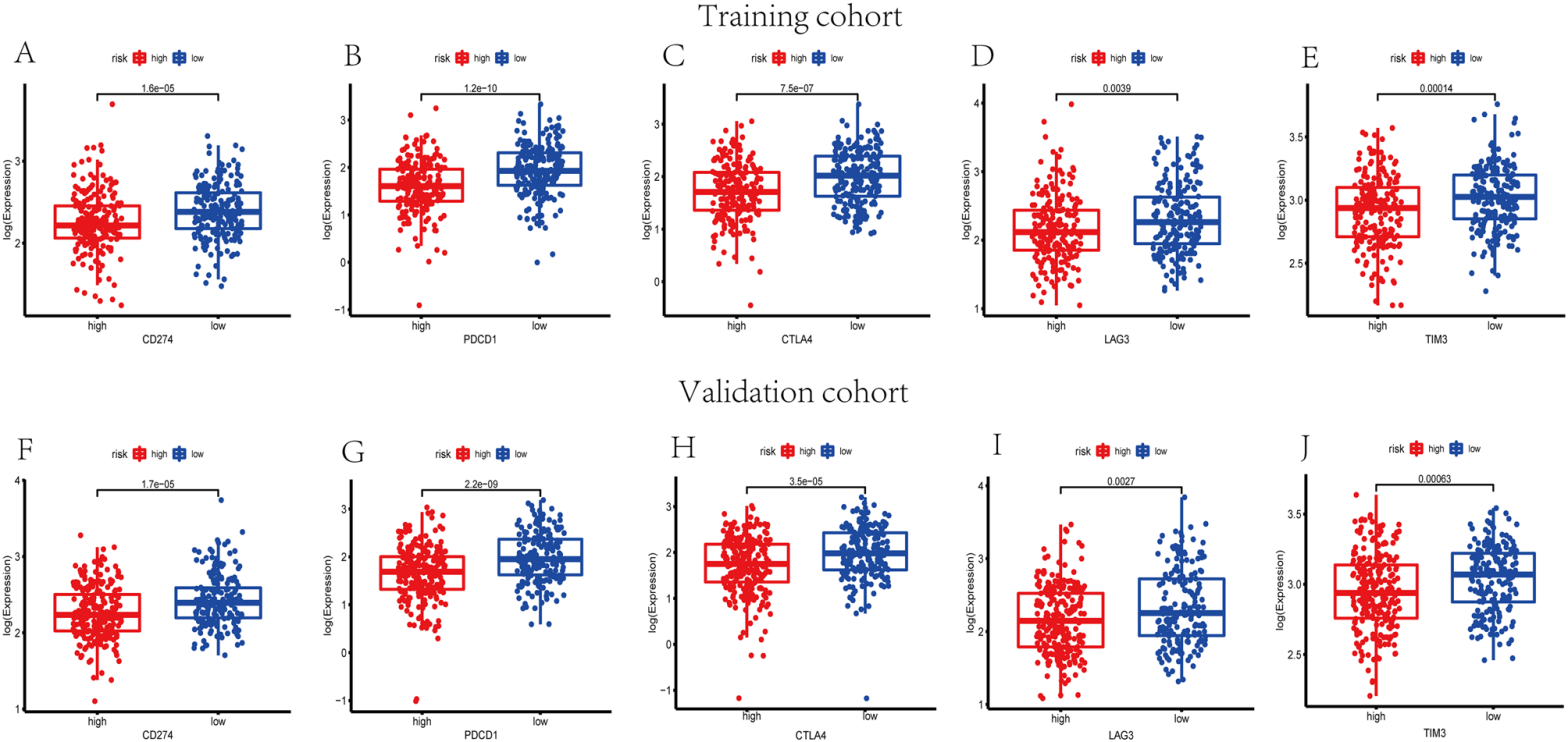
Immune checkpoint genes expression levels in high-risk group and low-risk group. The expression levels of *CD274* (**A**), *PDCD1* (**B**), *CTLA-4* (**C**), *LAG3* (**D**) and *TIM3* (**E**) of each group in training set (*p* < 0.01). The expression levels of *CD274* (**F**), *PDCD1* (**G**), *CTLA-4* (**H**), *LAG3* (**I**) and *TIM3* (**J**) of each group in validation set (*p* < 0.01).

**Figure 12:**
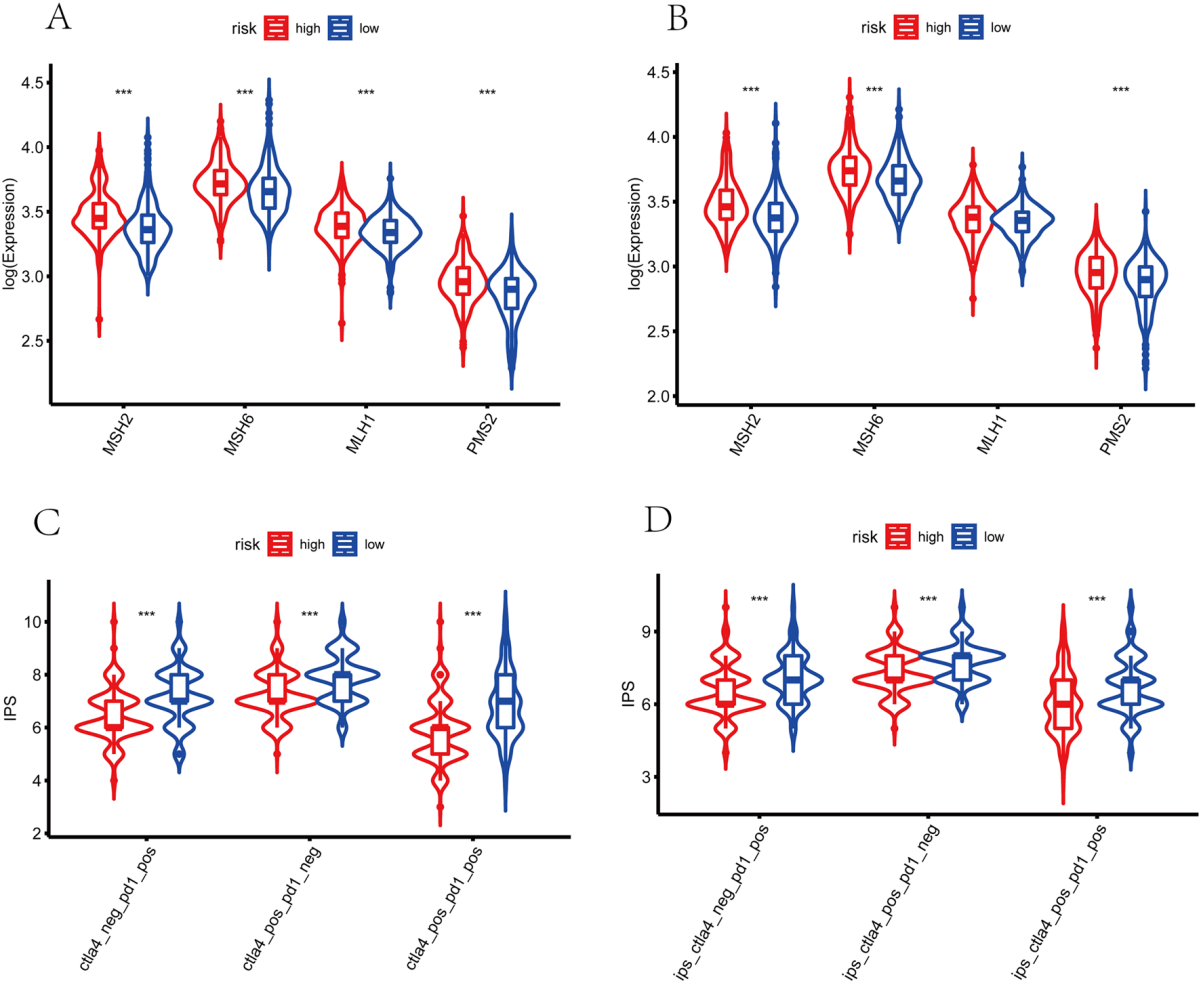
Prediction of immunotherapy response. The expression of mismatch repair genes in carcinoma tissues, MSH2, MSH6, MLH1, and PMS2, expressed remarkably lower in the low-risk sets in training cohort (**A**) and validation cohort (**B**). The IPS of anti-PD-1, anti-CTLA-4, and anti-(CTLA-4 plus PD-1) in the low-risk groups were significantly higher than those in the high-risk sets of training set (**C**) and validation set (**D**), indicating that patients with higher risk scores had worse immunotherapy responses.

To investigate the potential role of the exosome-associated-lncRNA risk signature in prediction of immunotherapy response, immune checkpoint blockade key molecules, microsatellite instable (MSI) in tumor tissue were further analyzed. Five vital immune checkpoint inhibitor genes were selected to explore the potential ability of the risk model to predict the response of ICB therapy. Figure 11 suggests that the expression levels of *CD274*, *PDCD1*, *LAG3*, *CTLA4* and TIM3 were higher in low-risk group among training and validation cohorts (*p* < 0.05). Subsequently, the transcriptional expression of obvious mismatch repair genes in tumor tissues were calculated, resulting that *MSH2*, *MSH6*, *MLH1*, and *PMS2* were all expressed markedly lower in the low-risk set (Fig. 12A,B), indicating that the microsatellites were more stable in the high-risk set. Eventually, the TCIA database was applied to calculate the IPS for each sample, which served as a favorable predictor of response to anti-CTLA4 and anti-PD-1. The IPS of anti-CTLA-4, anti-PD-1, and anti-CTLA-4 plus anti-PD-1 were higher in the low-risk group, which strongly forecasted that patients in low-risk group had better immunotherapy responses (Fig. 12C,D). Sum up, biomarkers mentioned above forecasted that patients with lower risk scores were probably to get better immunotherapy response.

## Discussion

As a highly heterogeneous disease^24^, BC is consisted of breast tumor cells as well as several categories of normal cells, for instant stromal cells, immune cells, and fibroblasts^25^. Despite of the development of multidisciplinary approaches, advanced BC patients with distant metastases are always considered remediless. Recently, immunotherapy provided the unprecedented opportunities to malignancies effective treatment and tumor immunology research has been the fastest growing area in cancer, including BC^26^. Exosomes are a kind of nanosized vesicles involved in varieties of cellular functions^14^, containing several proteins and nucleic acids including lncRNAs and miRNAs^27,28^. LncRNAs are a wide heterogeneous group defined as transcripts longer than 200 bp in length and lacking open reading frames^9,29^. Some of these lncRNAs are presented via genome-wide association studies and involved in several diseases including malignant tumors^9,29,30^. Additionally, some studies indicated that lncRNAs are critical modulators of cancer-associated signaling pathways and function as oncogenic lncRNAs or tumor suppressor^31–34^. Exosomal lncRNAs play an important role in cancer cell proliferation, angiogenesis, invasion, and drug resistance^9^. Koldemir and his colleagues reported that the expression of lncRNA *GAS5* in BC cells leaded to the enrichment of their exosomes, which was thought to be an apoptosis-inducing marker^35^. As a well-known lncRNA related to carcinoma progression, the high expression level of exosomal *MALAT1* induces tumor proliferation and migration in NSCLC and is closely associated with the metastasis of lymph node^17^. Besides, exosomal lncRNAs are also identified as potential biomarkers and therapeutic agents. As an oncogenic lncRNA, exosomal *H19* can be detected in bladder cancer patients’ serum and plasma, which has been considered as a highly potential cancer biomarker in bladder cancer^36^. Interestingly, a study in multiple myeloma (MM) verified that the transfer of exosomal *lncRUNX2-AS1* from MM cells to mesenchymal stem/stromal cells can inhibit the osteogenesis via the exosomal lncRUNX2-AS1/RUNX2 pathway^37^.

In current study, a dataset of lncRNA expression in BC samples from TCGA were analyzed and the prognostic lncRNAs related to exosomes were screened out. Subsequently, according to the relevance between the expression levels of lncRNAs and the OS, 31 exosome-associated lncRNAs were screened out as prognostic lncRNAs. Among them, 15 exosome-related lncRNAs were identified to construct a new prognostic risk model. The Kaplan–Meier curve suggested an obvious distinction in OS between the high-risk and low-risk sets. The AUCs of the lncRNA-based risk scores in training cohort for the 2-year, 3-year and 5-year OS prediction models were 0.798, 0.784 and 0.750, respectively. Besides, a lncRNA-mRNA co-expression network was constructed and the functional analysis was further conducted, proposing a high enrichment of immune-associated biological processes. Eventually, the infiltration of distinct immune cells in cancers were analyzed to further study the characteristics of TIME.

The 15 prognostic lncRNAs related to exosomes were composed of *MEF2C-AS1*, *SOCS3-DT*, *LINC01711*, *MRPL20-DT*, *LINC00702*, *MAL2-AS1*, *USP30-AS1*, *WASHC5-AS1*, *TBL1XR1-AS1*, *LINC02463*, *LINC02037*, *ZBTB20-AS1*, *BTBD9-AS1*, *SLC24A3-AS1*, *CACNA1C-IT3*. Recently, a study in cervical cancer suggested that overexpression of lncRNA *MEF2C-AS1* could suppress the growth, invasion and migration of tumor cells through inhibiting *miR-592* by targeting *RSPO1*^38^. In addition, Luo et al. discovered that the expression of *MEF2C-AS1* was markedly lower in the plasma of diffuse gastric cancer patients and the downregulation of *MEF2C-AS1* facilitated invasive tumor behaviors in in-vitro experiments^39^. Besides, *MEF2C-ASI* was identified as a prognosis-related biomarker to construct a lncRNA prognostic signature for predicting overall survival in elderly patients with BC^40^. These findings are consistent with our results, so we speculated that *MEF2C-AS1* might be a protective factor in tumor progression. As for lncRNA *LINC00702*, a study in malignant meningioma (MM) revealed that high expression level of *LINC00702* indicated poor prognosis. After that, *LINC00702* served as an oncogene in MM via modulating *miR-4652-3p/ZEB1* axis and activating Wnt/β-catenin signaling pathway^41^. Then, *LINC00702* was reported to accelerate the progression of ovarian cancer by acting with *EZH2* to repress *KLF2* transcription^42^. In our study, *LINC00702* was related to poor clinical outcome. On the other hand, *LINC00702* was found downregulated in colorectal cancer and repressed tumor cell proliferation, invasion, and migration through suppressing the PI3K/AKT pathway by promoting *PTEN* expression^43^. Besides, Yu et al. conducted an experiment in non-small cell lung cancer (NSCLC) to detect the role of *LINC00702* in tumor progression. The results indicated that overexpression of *LINC00702* significantly repressed growth and metastasis of NSCLC cells by inducing apoptosis in vivo and in vitro^44^. Vishnubalaji R et al. found that the expression of *LINC01711* was positively associated with the expression of TGFβ1 in a triple-negative breast cancer (TNBC) patient cohort^45^. Xu and colleagues performed experiments in esophageal squamous cell carcinoma (ESCC) to clarify the mechanism underlying the effect of *LINC01711* on its treatment and prognosis. The results indicated that high expression level of *LINC01711* was detected in ESCC tissues, which was related to poor prognosis. In addition, exosomal *LINC01711* promoted the progression of ESCC cells through upregulating *FSCN1* and downregulating *miR-326*, thereby facilitating the incidence and development of ESCC^46^. Moreover, the study conducted by Chen et al. verified in the first instance that high expression of *USP30-AS1* was closely related to poor prognostic outcomes of patients with cervical cancer^47^. Additionally, USP30-AS1 lncRNA was reported as a protective lncRNA in several studies to establish prognostic signatures for BC patients^48–50^. However, no research indicated the prognostic function in the other remaining lncRNAs in tumors. Hence, further studies were significant to clarify how these lncRNAs influence the prognosis of BC patients through TME.

Recently, several studies reported that some certain lncRNAs were selectively packaged into exosomes. They proposed that the abundance of exosomal RNA transcripts was closely related to their expression in the cell of origin. Unfortunately, the mechanism underlying the packaging of the contents with certain biological functional into exosomes was still not well understood nowadays^51^. In our study, functional enrichment analysis indicated that the prognostic exosome-related lncRNAs commonly enriched in the pathways associated to immunity. The infiltrating levels of some immune cells were elevated with the decreased risk score and some immune signatures were activated with the decreasted risk score. Based on published works, a growing number of studies focused on TIME identified the potential effects of lncRNAs on infiltrating immune cells. A study in hepatocellular carcinoma (HCC) demonstrated that HCC-derived exosomal lncRNA *TUC339* played an important role in macrophage activation and M1/M2 polarization, which clarified the complicated interactions between tumor and TME mediated by exosomal lncRNAs^52^. Moreover, Domvri et al. found that exosomal lncRNA *PCAT-1* had close connection with the immune response and tumor stroma remodeling. The results revealed that *PCAT-1* regulated Kras-associated pulmonary chemoresistance by increasing the expression of the immunosuppressive microRNAs miR-182/miR217 in lung tissues, further contributing to the formation of pre-metastatic niche and the subsequent burden of pulmonary metastasis^53^. Consequently, we inferred that the characteristics of infiltrating immune cells were closely related to the exosome-related lncRNAs and verified that exosome-related lncRNAs based on risk model could play an important role in immune cell infiltration.

Because of the results linked the exosomal lncRNA risk model to immune infiltration in BC, these exosome-related lncRNAs may be targets for treatments with immune checkpoint inhibitors. In our study, high-risk group based on 15-exosomal-lncRNA risk model presented lower expression levels of immune checkpoint molecules with poor clinical outcomes. Additionally, MSI analysis and IPS analysis were applied to patients in high- and low- risk groups, indicating that high-risk group patients tended to present worse immunotherapy response. Consequently, exosome-related lncRNAs signature could efficiently predict immunotherapy response in BC patients, which could be beneficial for the development of clinical treatment strategies.

This was the first study to build and validate an exosome-associated lncRNA signature based on 15 exosome-associated lncRNAs from a public database with retrospective data. And the signature was identified as an independent prognostic factor for BC patients. Nevertheless, some limitations in current study should be noticed. First, a single data source was used from TCGA database. Second, some prognostic factors such as chemotherapy data and immunotherapy data were not included in the univariate and multivariate COX regression analysis because of the incomplete data for these parameters. Third, there were seldom studies about BC involving the 15 selected lncRNAs. It is meaningful to conduct wet experiments to validate the expression of the selected lncRNAs in exosomes secreted from BC in the future. At last, our work only initially revealed the correlation between exosome-associated-lncRNA risk score and immune cells infiltration and immunotherapy response, but seldom involved the relationship between exosomal lncRNAs and TIME. Therefore, additional prospective studies are important to verify the value of this signature in prognosis and the underlying mechanisms of exosomal lncRNAs in anti-tumor immunity should be further detected by wet experiment. In general, the results of our study will provide novel ideas for BC treatment.

## Conclusion

A novel exosome-related lncRNA risk model was established through bioinformatics approaches and relevant algorithms, that was related to the immune cell infiltration. Our study indicated that the exosome-related lncRNA-based signature showed outstanding performance in determining the prognosis and immunotherapy responsiveness of BC patients. Moreover, it can be served as a potential independent prognostic factor and provide novel insights for immunotherapy for BC.

## Supplementary Information

Supplementary Table 1.

Supplementary Table 2.

Supplementary Table 3.

Supplementary Figure 1.

## Data Availability

The datasets analyzed during the current study are available in the TCGA database (http://cancergenome.nih.gov/).
