# Supplementary figures and images for: An exosome-related long non-coding RNAs risk model could predict survival outcomes in patients with breast cancer
Source: Sci Rep. 2022 Dec 24;12:22322. doi: 10.1038/s41598-022-26894-5 (PMC9789946; doi:10.1038/s41598-022-26894-5)

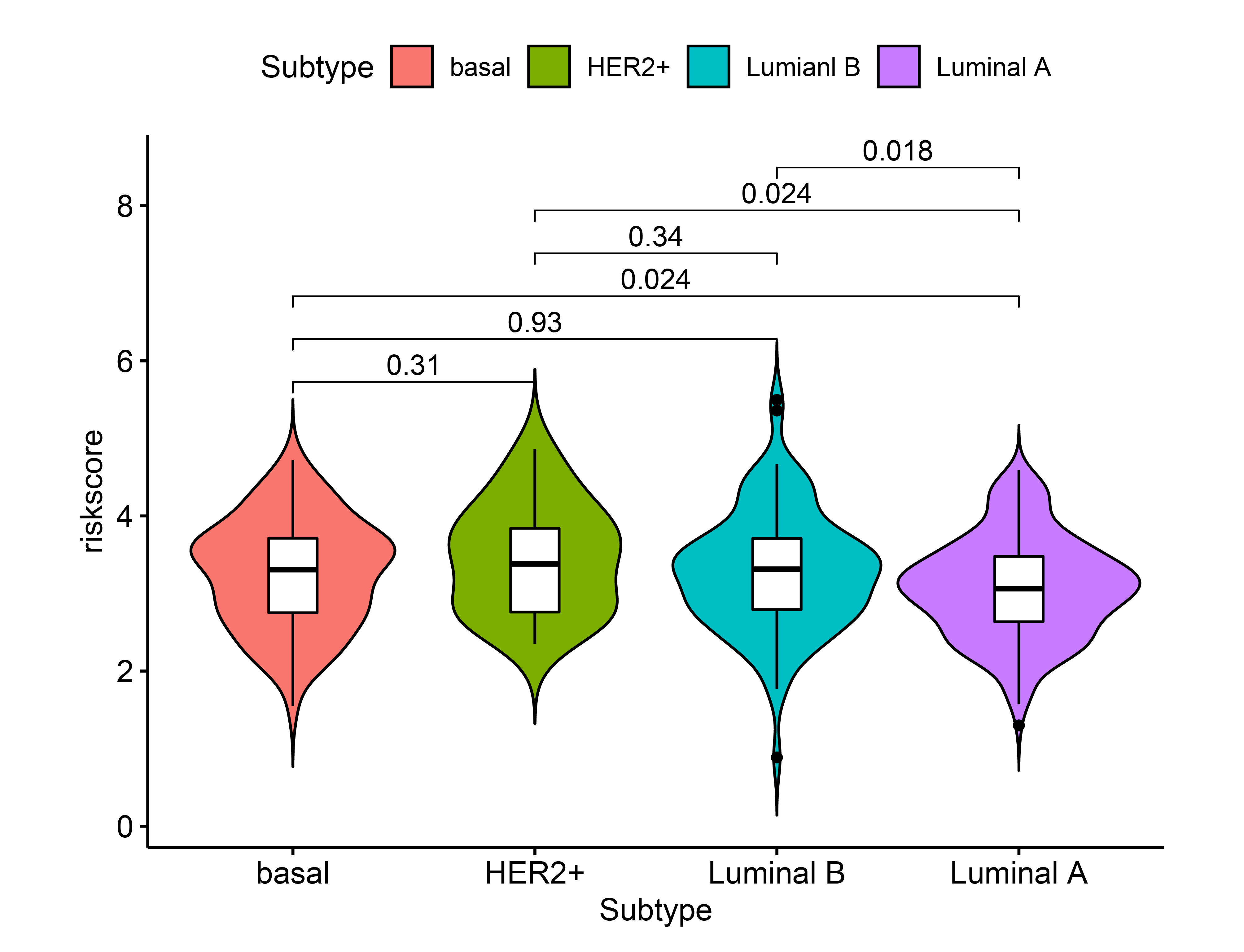

Supplement: Supplementary file 4 — Supplementary Figure 1. [file 41598_2022_26894_MOESM4_ESM.tif]
